# Supplementary figures and images for: The NKG2D ligand ULBP4 is not expressed by human monocytes
Source: PLoS One. 2021 Feb 8;16(2):e0246726. doi: 10.1371/journal.pone.0246726 (PMC7870063; doi:10.1371/journal.pone.0246726)

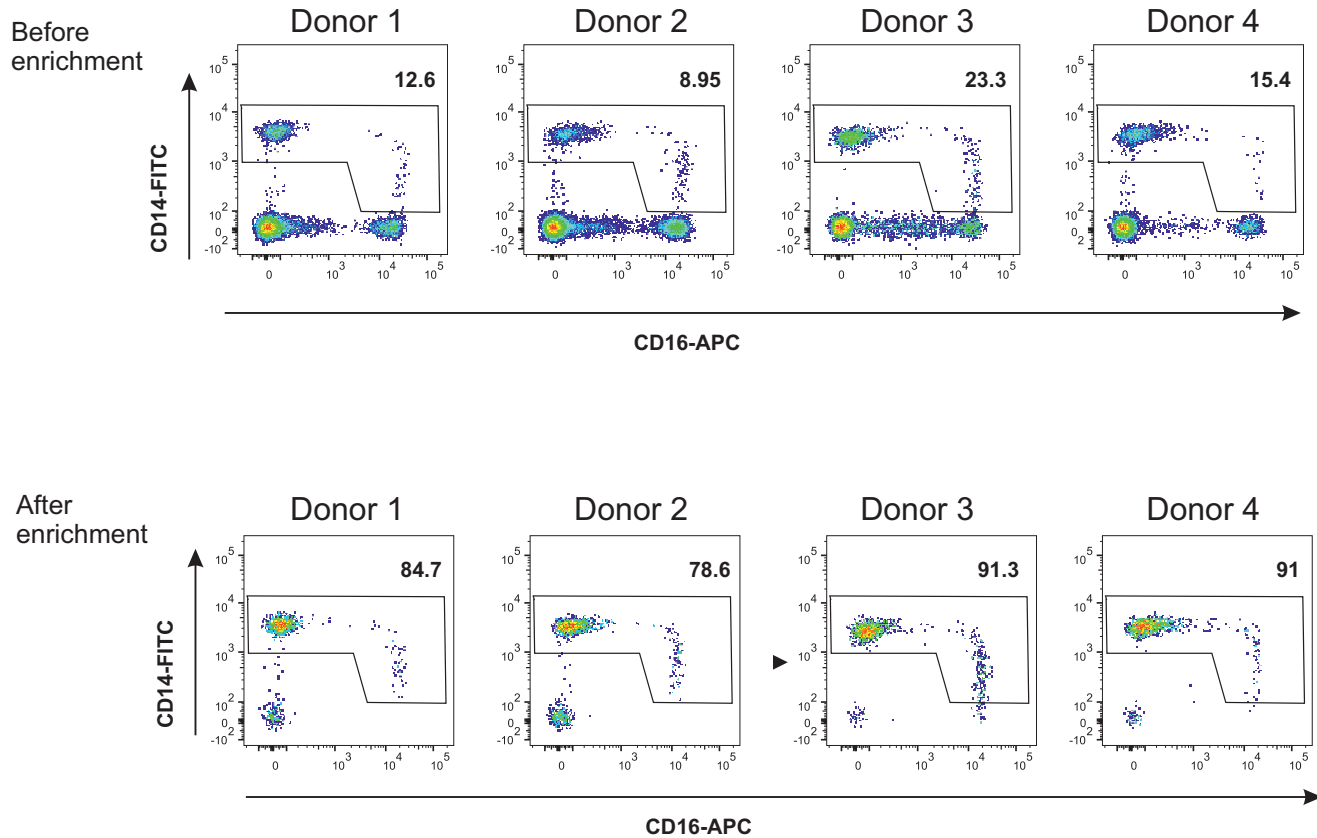

Supplement: S1 Fig — Monocytes enriched from PBMC using a pan monocyte isolation kit were directly analysed by flow cytometry to validate the enrichment. All three major blood monocyte populations (classical (CD14highCD16–), non-classical (CD14lowCD16+) and intermediate (CD14highCD16+) monocytes) were successfully enriched. The percentage of total monocytes is indicated. (PDF) [file pone.0246726.s001.pdf]

## LPS-treated monocytes

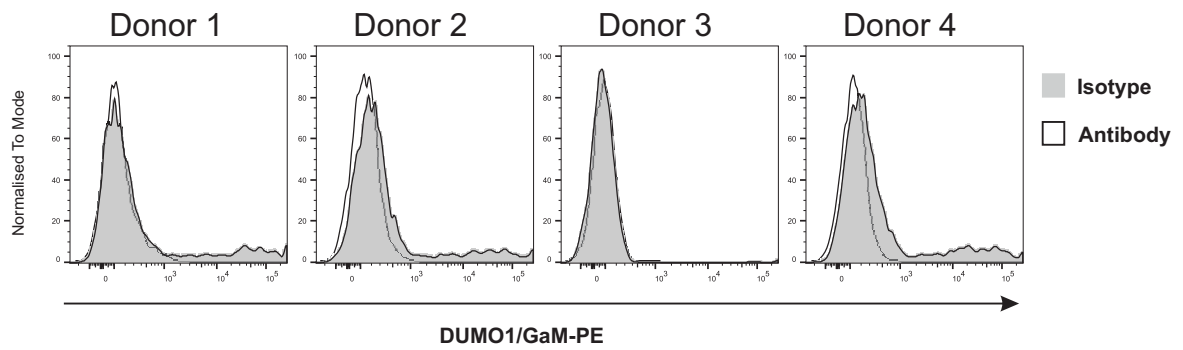

## poly(I:C)-treated monocytes

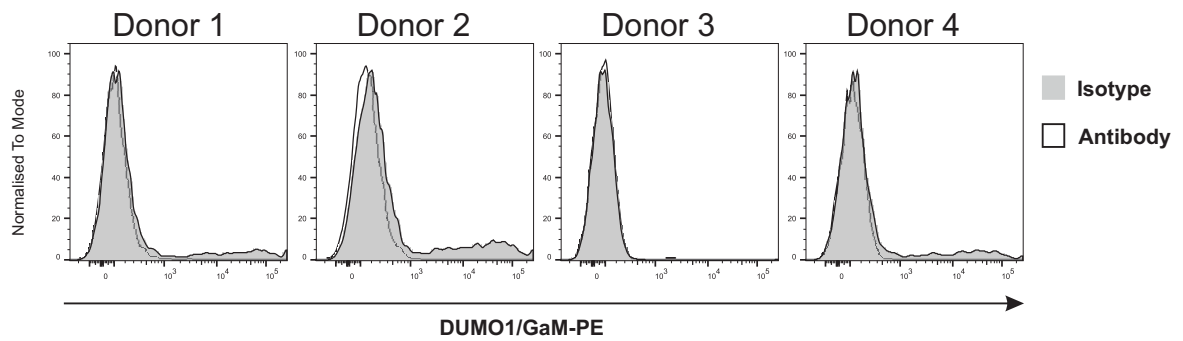

Supplement: S2 Fig — Monocytes enriched from PBMC were cultured for 16 h in the presence of lipopolysaccharide (LPS) (at 500 ng/mL) or with polyinosinic:polycytidylic acid (poly(I:C) (at 10 μg/mL) and subsequently analyzed by flow cytometry for ULBP4 surface expression with DUMO1. Overlays of DUMO1 stainings (solid lines) with isotype controls (gray filled) of CD14+ monocytes are shown for all four donors. Note that isotype controls, but not DUMO1, unspecifically stained a minor portion of cells. (PDF) [file pone.0246726.s002.pdf]
